# Romantic racism: A reassessment of Carl Gustav Carus’s writings on race and human inequality

**DOI:** 10.1017/mdh.2025.8

**Published:** 2025-04

**Authors:** Stephan Strunz, Marina Lienert, Florian Bruns

**Affiliations:** Institut für Geschichte der Medizin, Technische Universität Dresden, Dresden, Germany

**Keywords:** Racism, Romantic medicine, Anthropology, Craniology, Germany, International

## Abstract

This paper aims to provide the first comprehensive evaluation of Carl Gustav Carus’s writings on race and human inequality. We demonstrate that Carus, an eminent nineteenth-century physician emblematic of romantic medicine, was deeply engrossed in racial science, exploring anatomical, anthropological, and craniological dimensions of race across no less than twenty-five works spanning three decades. Carus’s engagement with race stemmed from *naturphilosophisch* anatomical and physiological considerations, which evolved into physiognomic and psychological inquiries. While previous research has construed Carus as a precursor of Arthur de Gobineau, we argue that he was intellectually much more closely aligned with the ‘American School’ of ethnology, represented by figures such as Samuel G. Morton, George R. Gliddon, and Josiah C. Nott. Closely monitoring international discourses of scientific racism, Carus sought to propagate these notions among German readers and position himself within international debates. The international reception, however, was limited by the Romantic framework of Carus’s scientific racism, which was unintelligible to contemporaries. While sharing an implicit methodological bias with Morton and his followers, affirming white superiority and legitimising colonisation, the Romantic underpinning of his race treatises made it difficult for mid-nineteenth-century race theorists to fully endorse him. Nonetheless, Carus, often lauded as polymath with a humanistic orientation, besides his achievements, helped to create a theoretical basis for the othering and dehumanisation of large parts of the global population.

When the Black American tragedian Ira Frederick Aldridge and his troupe stopped in Dresden in 1853, the city’s foremost physician, Carl Gustav Carus, was eager to witness the spectacle. While the press, public, and even the royal family applauded the troupe’s performances of Shakespeare,^1^ Carus had a peculiar fascination with Aldridge’s appearances on stage. Having dabbled in race science and craniology the previous fifteen years, Carus saw Aldridge’s portrayal of Othello as a window into the true essence of the eponymous play. ‘Othello’s fiercely good-natured and yet unstable love, but also his easy infatuation and his ultimate outburst of rage’, could only be effectively conveyed by a performer with ‘a receding forehead like that of a true Negro,’ a physiognomic feature he considered indicative of racial inferiority. Conversely, he believed that ‘well-educated’ and ‘higher natures’ embodied by white performers would fail to bring out the character’s intellectual simplicity as well as his uncontrollable affect.^2^ For this reason, Carus desired nothing more than to obtain a plaster cast of Aldridge’s head to add to his craniological collection.^3^

As anecdotal as this incident may seem, Carus’s encounter with Ira Aldridge is emblematic of his larger obsession with racial classification and white supremacy, a trait that would firmly link him to contemporary protagonists of scientific racism such as physician and naturalist Samuel George Morton, the Egyptologist George Robbins Gliddon, and the physician and anthropologist Josiah C. Nott. Unlike these figures, Carus has not been considered a major player in nineteenth-century discourses of scientific racism. Instead, scholars regard him as one of the most important proponents of *Naturphilosophie* and an eminent authority in romantic medicine.^4^ In medical historiography, Carus has been hailed for his contributions to gynaecology, psychology, and medical reform.^5^ With the rise of scientific medicine, Carus’s understanding of anatomy, physiology, and anthropology went quickly out of fashion from the 1830s onwards.^6^ His writings on race, however, continued to attract the interest of like-minded thinkers, the most prominent among them being race theorist Arthur de Gobineau. At the beginning of the twentieth century, Carus experienced a renaissance both among supporters of German Neo-Romanticism like Ricarda Huch or Theodor Lessing, and exponents of characterology like Ludwig Klages. Many of Carus’s works were reprinted in the 1920s and 1930s when racial research was very much in vogue in Germany.^7^ Although this line of reception is generally considered a posthumous development,^8^ it is important to note that Carus paved the way for it by closely affiliating himself with the most outspoken proponents of scientific racism during his lifetime.

In this paper, we will reassess Carus’s writings on race and human inequality, elucidating the deeply ingrained racial bias present in his works. Carus positioned himself deliberately within debates on racial classification, craniological sciences, and comparative anatomy, with the hopes of reaching an international readership. This, in turn, led several proponents of scientific racism to cite his work. Although many scholars have noted Carus’s preoccupation with race, a comprehensive analysis of his writings on the subject has yet to be undertaken. This lacuna may be attributed to the fact that Carus’s works, deeply rooted in German *Naturphilosophie*, never gained comparable prominence within the international research community. A prolific author, painter, and polymath, he wrote on a broad and heterogeneous range of subjects, from anatomy, gynaecology, metaphysics, physiology, and psychology to anthropology, aesthetic theory, metaphysics, and zoology.

Previous research has, for the most part, pinpointed and examined Carus’s racial theories mainly in two later works: a festschrift on the occasion of Goethe’s one hundredth birthday, *On the Unequal Capacity of the Different Human Races for Higher Intellectual Development* (1848)^9^, and a physiognomic treatise, *Symbology of the Human Figure.* ^10^ Scholars have argued that Carus’s romantic *Naturphilosophie* led him to conceive of the world in clear-cut polarities. Carus believed that racial classification would align with the sun’s passage over the earth, following the cycle of eastern twilight, day, western twilight, and night.^11^ Others have scrutinised the relationship between Carus’s craniological works and race-related questions, highlighting its indebtedness to physiognomy and the logistical networks of skull collection.^12^ Analysing Carus’s physiognomic works, Jutta Müller-Tamm has found that the shape of the limbs symbolised an individual’s physical constitution, while facial features were thought to reveal temperament. Of all the bones, the cranium was deemed the most critical since it divulged a person’s intellectual abilities.^13^ As Michael Hagner has observed, Carus’s racial physiognomy overlapped with a disdain for women and the disabled, as well as a romantic veneration of ‘geniuses.’^14^ As for so many of his contemporaries,^15^ a deeply ingrained racial bias motivated his endeavours in racial classification, which tended to align his metric findings with stereotypical beliefs about non-Europeans.^16^

In the following, we will make four major points. First, significantly broadening the scope of our analysis, we will briefly outline the trajectory of Carus’s theory of race, which pervades and connects a much larger body of works from 1835 until the late 1860s.^17^ Second, as Carus’s engagement with racial theory emerged from anatomical and physiological questions, we will argue that it was not physiognomy^18^ but *naturphilosophisch* comparative anatomy that provided the foundations for his scientific racism. Third, we will demonstrate how Carus shaped this anatomical foundation into a physiology of race that integrated selective empirical observations, aesthetic judgements, and historical speculation with romantic metaphysics. His physiology of race evolved into an anthropological project that would restrict the romantic notion of universal humanism to a small portion of the global population. Fourth, we will trace Carus’s attempts to engage with international discourses of scientific racism and reconstruct the reception of his racial theories. Carus read and cited major figures in craniology and early anthropology and sought their attention. Facing criticism for his *naturphilosophisch* approach by European anatomists, Carus turned to the ‘American School’ of Ethnology in the hopes of a more favourable reception. Ultimately, however, the influence of Carus’s theory of race was limited by the romantic framework in which it operated.

## Carus’s views on race: A trajectory

Carus’s preoccupation with race spans a period of more than thirty years. During this time, he refined and reconceptualised important aspects of his theory, putting it into dialogue with works by other racial scientists. In re-examining Carus’s writings, we identified a total of at least twenty-five publications, including two second editions, in which he considered race-related questions. The first approach he took to race was motivated by an engagement with comparative anatomy and physiology. The origins of his theory of race trace back to a treatise entitled *System of Physiology* from 1838 in which he outlined a general theory of biological *Entwicklungsgeschichte* (*Developmental History*).^19^ The first volume contained a lengthy section on racial classification that correlated his categorisation of humankind with the solar cycle. Carus referenced the racial taxonomies of Johann Friedrich Blumenbach, Jean-Baptiste Bory de Saint-Vincent, and Rudolf Wagner but noted that their systems failed to identify ‘a higher level of reason.’^20^ Carus found such a higher level of reason in the correspondence of four races to four realms of nature and four solar stages of the earth, diving humanity into ‘people of the night’ (sub-Saharan Africans, African Americans), ‘people of the Eastern twilight’ (Chinese, Indians, Malay), ‘people of the Western twilight’ (Indigenous Americans), and ‘people of the daylight’ (Europeans). The ascent of the sun was thus reflected in the ascent of human civilisation, which found its zenith in Europe, whose inhabitants Carus identified according to Blumenbach’s definition of ‘Caucasians’.^21^

This crude cosmological classification was soon bolstered by anatomic evidence, in particular by measurements of the skulls of various people. Here, too, Carus’s first contribution was outlined in *System of Physiology*, in its third volume, published in 1840. He would go on to publish several illustrated atlases of craniology,^22^ as well as a handbook in which he explained his method of measuring the skull as opposed to the phrenological competition.^23^ In the mid-1840s, he expanded this argument to other parts of the body and considered the human shape (*Gestalt*) in general as indicative of various racial characteristics. Hands, for instance, were thought to be expressive of sensitivity; white skin allowed for a refined sensitivity, whereas the pigmented hands of Mongols and Native Americans were destined for constant movement.^24^ In the late 1850s, he undertook a meticulous examination of contemporary racial science and positioned himself within that discourse with a review essay.^25^ Considerations of the anatomical-philosophical foundations of racial classification also recur in his late work, *Nature and Idea* (1861).^26^

By the end of the 1840s, historical and psychological arguments had entered into Carus’s racial theory. This stance is epitomised in his primary treatise on race, *On the Unequal Capacity of the Different Races.* Where his previous works on race had relied chiefly on anatomical, physiognomic, and *naturphilosophisch* considerations, Carus now employed many historical examples to create psychological archetypes for each of the races. After reiterating his solar taxonomy of 1838, Carus delivered a detailed description of each of the four races, grounded in general historical trends, which reproduced common Eurocentrist narratives. According to Carus, the history of slavery was a significant indicator of the intellectual inadequacy of Black people. Despite his opposition to slavery, Carus contended that its persistence among Black people indicated their ‘lack of even a basic sense of freedom.’^27^ Carus claimed that only the most oppressive form of slavery instilled a thirst for rebellion among descendants of sub-Saharan Africans, pointing to the struggle for independence in Haiti.^28^ Subsequently, Carus argued for white superiority along similar lines. He portrayed European colonialism as an epochal event made possible by the ‘highest power of the mind.’ Only ‘the most powerful centre of the true daylight peoples – Romanics (Spaniards) and Iberians …, Celts and especially Germanics (English)’ possessed enough mental fortitude to ‘pour forth a new population across the corners of the globe.’^29^

Carus’s final approach to race can be described as biographical or anecdotal. He developed these thoughts primarily in his autobiographical work *Memoirs and Memorabilia*, as well as in his letters and travelogues.^30^ His remarks on race occupy only a minor portion of his recollections as a whole, but even when Carus encountered people and objects that might have challenged the views of others, he found reasons to maintain his belief in racial inferiority. For example, when confronted with Chinese artisanship in London, Carus could not help but view it as ‘junk’ that could never compete with ‘the pure form of the Sistine Madonna.’ Chinese books, weapons, furniture, jewellery, porcelain, coins, ship models, and other artifacts served only as testimony to him that the ‘light of higher beauty has never shone on a nation of more than 300 million people.’^31^

## A foundation in comparative anatomy

In the following, we will situate Carus’s race theory within the broader context of his *naturphilosophisch* works on comparative anatomy. For Carus, as for many naturalists of the Romantic era, comparative anatomy and physiology were mutually dependent, as morphological phenomena were directly related to organic functions.^32^ To trace the racialisation of his thought, we place the *System of Physiology*, in which his theory of race first emerged, in relation to two early works on comparative anatomy, one concerning brain development in animals and humans and another comparing skeletal development across species.^33^

Interestingly, his first published work, a treatise on brain development, made no mention of race. Drawing on the conceptual arsenal of Johann Gottfried Herder and Friedrich Wilhelm Schelling, Carus believed that an absolute principle or ‘highest idea’ (*höchste Idee*) of organic development was manifest in the ‘perfection’ (*Vollkommenheit*) of man’s cerebral ‘formation’ *(Bildung*).^34^ Carus identified a series of morphological relationships that demonstrated this perfection, particularly ‘the extent [*Maß*] to which the cerebellum appears subordinate to the hemispheres and the spinal cord to the cerebellum’, in both quantity and quality.^35^ He argued that in human beings, the hemispheres were ‘extraordinarily large in comparison to the other parts of the brain,’ representing the ‘noblest organic form, the sphere.’^36^ The perceived perfection of the hemispheres was thus rooted in an aesthetic judgment – the divine spherical form, which he regarded as superior to more angular, terrestrial shapes.

In 1814, in order to mark a strong contrast with Gall’s phrenology, the young Carus cautioned against correlating mental capacities with cerebral structures, emphasising that ‘inner freedom and external circumstances’^37^ played a more important role in psychical development. Since the perfection of the human hemispheres symbolised the unity and ‘ideal of the species’^38^ (*Ideal der Gattung*), Carus likely saw no need for racial differentiation at this stage. This view aligned with the opinions of Herder and Schelling, neither of whom considered race a factor in the development of species.^39^ Nevertheless, a closer reading shows that the germ of rupture was already present. For Carus, the notion of species, when applied to living beings, enabled the observation of various degrees of approximation to the ideal form. If parts of an individual’s brain were ‘imperfectly or partially developed,’ this would lead to an ‘inclination [*Hinneigen*] towards an animalistic character.’^40^ Smaller hemispheres, for instance, would result in a lesser predisposition [*Anlage*] toward reflection, whereas a stronger spinal cord would increase the inclination toward energetic or impulsive nervous reactions.^41^ Thirty years later, Carus would develop this concept of cerebral inclinations into a theory of racial differentiation, ultimately positing that certain races were inherently incapable of approaching the species ideal.

The second work in which Carus laid out the foundation of his theory of race was published nearly fifteen years later, in 1828. *On the Primordial Elements of the Skeleton and the Shell* ^42^ linked his theory of cerebral development with the anatomy of the skull. This treatise was heavily influenced by the ‘osteological programme’^43^ of Johann Wolfgang von Goethe and Lorenz Oken. Goethe had introduced the concept of the archetype (*Urtyp*) to account for common osteological forms across all living organisms.^44^ In the 1790s, his quest for these fundamental forms led him to study animal vertebrae, in which he sought ‘an *inclusive form*, a pattern that would contain all of the parts really exhibited by the range of different vertebrate species.’^45^ Both Goethe and Oken proposed that the bones of the skull could be regarded as more advanced vertebrae, offering a new framework for the analysis of archetypal structures.^46^

Building on Goethe’s theory of six archetypal cranial vertebrae, Carus conducted his own comparative anatomical studies. In 1828, he repeated his claim that the human brain was the most perfect among all species, now tying its phylogenetic development to the formation of the cranial vertebrae. The gradual progression of the six archetypal plates of the cranium progressed from jawless fish via the skulls of intermediate animals, ‘to the plates forming the braincase and face of the most advanced creature, man.’^47^ He argued that the brain’s three major masses (the cerebellum, corpus quadrigemina, and hemispheres) were associated with three skull vertebrae (hinder, central, frontal) and that each related to a specific sense (hearing, seeing, smelling) and unspecified mental capacities. For future works, he envisioned a deeper engagement with these mental capacities that would undoubtedly ‘pave the way for a scientific cranioscopy.’^48^

However, in 1828, race was still not in the picture. For the racialisation of his anatomical thinking, an external stimulus was needed. Indeed, the first mention of race in his works concerns a visit to Georges Cuvier’s *galérie d’anatomie comparée du muséum national* in 1835, where he was granted access to a private exhibition chamber containing the human remains of Sarah Baartman, a cast of Franz Joseph Gall’s skull, and the ‘curious squashed skulls of ancient Peruvian mountain dwellers.’^49^ On the way back to Dresden, he stopped in Göttingen to visit Blumenbach and his craniological collection. Glancing at the skulls of ancient Greeks, Jews, Peruvians, Caribbeans, and Malays, he was amazed at how ‘perfectly our unique formation [*Gestaltung*] is expressed in the skeleton.’^50^ Shortly after this trip, in 1837, Carus established his own craniological collection, starting with a plaster cast of what he believed to be the skull of Friedrich Schiller.^51^ The establishment of his craniological collection coincided precisely with his conceptualisation of race in *System of Physiology* in 1838.

## Romantic racial physiology: from anatomy to history

A first fragment of the *System of Physiology* was published in the philosophical-theological journal *Zeitschrift für Philosophie und speculative Theologie* as ‘On the life of mankind’, a title not only evoking Herder’s *Ideas on a Philosophy of Mankind* but also indicating that his physiology aimed at larger anthropological questions.^52^ Following Herder’s fundamental claim that ‘the human race on earth is one and the same species,’^53^ Carus asserts that ‘humanity is a realm of its own, a class of its own, an order of its own, a genus and a species of its own.’^54^ But where Herder had supported a strong monogenist position, arguing that there were no races but ‘only shades of the same great portrait that spreads across time and space,’^55^ Carus asserted the necessity of internal racial differentiation.

As Nicolaas Rupke has shown, Carus maintained the unity of mankind as a species but held that its various tribes or races *(Stämme*) originated ‘in their own province of distribution,’^56^ an ultimately polygenist theory of racial difference. His polygenism was coupled with the teleological concepts of his comparative anatomy. Since ‘any organism … must be considered not in its undeveloped state, but in its fully developed and completed form,’ it would be inappropriate to assess the position of humanity in the world ‘by looking at the Hottentot living in misery, or the Cretin, or the lost, feral, brutish human being in the wastelands.’ Instead, one had to consider the closest approximation of the ideal form, ‘the heights of humanity’ embodied by the geniuses of Europe.^57^

In the first volume of the *System of Physiology*, published in 1838, Carus integrated his fourfold racial taxonomy into a larger taxonomic system encompassing the entire natural realm. His racial science was embedded in a general theory of organic development, according to which the natural world gradually developed from the simple to the complex, from the liquid to the solid, and from the raw to the refined. Only after the land and the fauna had reached a certain degree of refinement – Carus mentions the ‘Diluvial period’ – was the stage set for the ascent of humanity, which then emerged spontaneously in diverse forms from primordial vesicles (*Urbläschen*).^58^ Race was the primary marker of human diversity, reflecting the history of organic development from simple to complex forms in the passage from ‘people of the night’ towards ‘people of the day’.^59^

For Carus, as for many others, skin colour was an important indicator of racial differentiation. When studying the skin of individuals referred to as ‘people of the night,’ Carus identified various shades of Black, associating them with stereotypical sensory characteristics: ‘The dark, nocturnal colouration is characteristic of all of them…. Everywhere strong charred secretions and strong skin exhalation.’^60^ Here, Carus relied on assumptions initially popularised by Immanuel Kant. Drawing on the phlogiston theory, Kant held that the skin of people of African descent contained elements of charcoal, resulting in its Blackness and, consequently, its inferiority to the skin of white individuals.^61^ Carus attempted to incorporate this idea into his theory of solar analogisms, connecting the supposed exhalation of the skin of Black people to ‘the stronger exhalation of carbon dioxide by plants during the night.’^62^ Indeed, Carus went a step further, seeking to corroborate phlogiston theory through his anatomical observations.

‘The carbonised secretions in the skin set in already during the life of the foetus - I have before me a four-month old Negro embryo upon which the dark colouration of the lower legs and genitals has already begun. The hair is also full of carbonaceous deposits and spirally coiled to the utmost extent (woolly hair).’^63^

We could not determine where Carus might have obtained this embryo. However, the mere fact that he examined the foetus of a Black child demonstrates his effort to establish an ontogenetic foundation for his theory of race. As bizarre as his attribution of carbonisation to various parts of the body may sound, it underscores how Carus’s racist assumptions were not discouraged but reinforced by selective case studies. Analysing the skin and skeletons of Black people only served to confirm his belief that ‘these are still distant proximations of the animal form.’^64^ A closer reading of Carus’s writings on anatomy shows his belief that the ideal form of humankind could only be conceived through the circular logic of differentiation. Being non-white was taken as evidence of a deviation from the ideal type, and the ideal type was measured by the degree of its non-deviation. In his *System of Physiology*, he explicitly states that features that were essentially non-Black characterised the purity of the anatomic form. Insofar as a ‘more or less purely white, or rather a purely translucent organisation of the skin, and less wooly … hair’ demonstrated ‘a truly human organisation,’ the ideal type of anatomy was ‘sufficiently characterised by the mere absence of any deviation (directed, in most cases, towards animal nature).’^65^ Carus thus acknowledged that there was nothing intrinsic to the anatomy of white people from the anatomy of non-whites. In Carus’s thinking, though certainly not in his alone, whiteness could emerge as the norm or ideal only when juxtaposed with some absolute other.^66^

In 1840, with the publication of the third volume of his *System of Physiology*, Carus finally connected his theory of race to his earlier studies on brain development and cranial morphology. As we have seen, Carus had long envisioned a ‘cranioscopy’ deserving of the title. Although the term was originally coined as a synonym for Gall’s phrenological project,^67^ Carus sought to distinguish his ‘new cranioscopy,’ intending to counter Gall’s ‘untenable hypotheses, … reveries and delusions.’ Consistent with his theory of inclinations and predispositions, Carus argued that the form of the skull indicated the ‘fundamental orientations of the soul’^68^ rather than specific moral qualities, as Gall had suggested. Citing experiments by the physiologists Jean Pierre Flourens, Heinrich Hertwig, François Magendie and others, Carus believed he had sufficient evidence to assign the mental faculties of will and sexual drive to the cerebellum, of feeling to the corpora quadrigemina, and of intellect to the hemispheres.^69^ The power of these faculties was then measured by the extent of the corresponding cranial vertebrae.

Drawing from samples of his expanding collection of skulls, he sought to demonstrate the predominance of the forehead and a predisposition toward higher intellect in ‘people of the day’, the predominance of the occiput and instinct-driven behaviour in ‘people of the night’, and the predominance of the central crown and a predisposition for feeling in Mongols, Malays and Americans.^70^ Although Carus still claimed that the association between cranial morphology and mental faculties resulted in mere predispositions, he categorically denied the possibility of Black people attaining higher intelligence.^71^ White people, on the other hand, epitomised for Carus the height of human civilisation since Greek antiquity. In this assessment, Carus fused two distinct but widespread aesthetic judgements. Following the Göttingen naturalist Blumenbach, Carus aestheticised the roundness of skulls; however, unlike him, Carus used these judgments to establish a hierarchy of mental superiority.^72^ Carus believed that skulls of the Pelasgian tribes, along with other Caucasian tribes, possessed a geometrical form which tended towards a golden equilibrium. By way of example, Carus pointed to both Pieter Camper’s ‘normal face angle of 90 degrees’ and a ‘beautiful ancient Greek skull in Blumenbach’s collection.’^73^ On this basis, Carus considered both the Grecian face angle and the roundness of ancient Greek skulls as epitomising a ‘purity of form’ (*Formenreinheit*) that the ‘other Caucasian tribes have not preserved.’^74^

These attributions were highly arbitrary, as evidenced by an example that Carus found particularly illuminating – the skull of Friedrich Schiller. In his 1843 *Atlas of Cranioscopy*, Carus had depicted a skull which he believed to belong to the great German poet, describing it as beautifully shaped and close to perfection: ‘Each of the three main vertebrae appears in full beautiful development; the central crown is particularly large, beautifully rounded and finely modelled.’^75^ As it turned out, Carus had not only mistaken another person’s skull for Schiller’s,^76^ but had also failed to compare the skull’s measurements with other data. The anatomist Hermann Welcker debunked the skull myth in 1883, proving decisively that it could not belong to Schiller. When Welcker visited Carus in 1862, requesting permission to compare the plaster cast of the skull with Schiller’s death mask, Carus approached him ‘with a mysterious look,’ cautioning him that ‘[t]he skulls are round; they do not want to be measured.’^77^ Had Carus ever measured the death mask of Schiller and compared the measurements with the skull, he would have noticed the discrepancy.^78^ Despite Carus’s claims of relying on sound measurements in his craniological works, his strong bias towards the supposed genius of the white race prevented him from assessing anything other than anatomical perfection.

Another ‘physiological interest’^79^ which he hoped would support his racial theories was the history of epidemics. Carus strongly believed that his fourfold racial categorisation was not only evident in the geographical distribution of people around the globe but also their chronological succession. The cradle of civilisation was to be found among the ‘people of the eastern twilight,’ from which it ‘only then spread, but with greater sophistication, to the people of the day, and ultimately, to the people of the western twilight.’^80^ Black people were omitted from the progress of civilisation altogether. Similarly, Carus did not conceive of the spread of cultural achievements as a linear process but as an arc in which only one race, the Caucasians, reached the peak of civilisation. Mirroring a common white supremacist narrative, indigenous Americans were considered to be cultured but ultimately doomed since Europeans had ‘taken possession of their land to such an extent … that we will experience the gradual extinction of the indigenous inhabitants of America.’^81^ The history of epidemics followed the same trajectory, never reversing its course. Ignoring the case of syphilis, Carus was convinced that the ‘movement and further development of such parasitic organisms in this direction’ could neither be ‘unusual’ nor ‘unexpected’ because it followed the same pattern as the development of human races.^82^ As we have demonstrated, Carus did not hesitate to consider select medical facts for the theory of race. In each instance, however, he twisted them to fit into larger metaphysical patterns.

Unsurprisingly, such characterisations rendered it easy to legitimise European colonisation or slavery, enabling Carus to extend his physiology into historical and geographical dimensions. As Carus put it, the colonisation of Africa and the Americas by white people not only demonstrated ‘the highest intellectual power of mankind,’^83^ but it was a demographic necessity. In an unpublished manuscript on the ‘physiology of the state’ (1850), Carus indulged in neo-Malthusian reflections. Since industrialisation led to a disparity between population growth and agricultural production, European states could not help but colonise non-European peripheries.^84^ Accordingly, the Atlantic slave trade did not amount to genocidal violence but corresponded to the natural organisation of Black people, who had been living in ‘reciprocal slavery, … often enjoying their condition as a dull form of happiness.’^85^ In a world characterised by insurmountable developmental gaps, racial inequality served as a means to justify the rule of one race over all the others. Carus constructed a system in which a physical and intellectual superiority that was already confirmed by history was legible in the slightest anatomical differences.

To summarise, Romantic *Naturphilosophie* deeply informed Carus’s theory of race. As a contemporary reviewer aptly noted, Carus’s physiology ultimately amounted to an anthropology grounded in comparative anatomy.^86^ Carus held that the phylogenetic development of body parts, particularly the brain and the skull, followed a teleological trajectory of gradual perfection *(Vervollkommnung*). Crania and brains expressed morphological perfection not only in terms of mass and volume but also geometrically. In man, the hemispheres and the skull vertebrae approached the mathematical ideal of the sphere. This framework enabled Carus to compare samples of various races with a fixed ideal, establishing ‘predispositions’ *(Anlagen*) and ‘inclinations’ (*Neigungen*) to varying degrees of human perfection. Although he did not initially associate morphological perfection with race, his increasing focus on craniology after 1835 led him to link race, cranial form, and mental faculties. Large and rounded European skulls were now seen as real-world approximations of the species ideal. Reflecting his polygenist framework, Carus increasingly reinterpreted ‘predispositions’ and ‘inclinations’ toward higher or lower intellectual development as insurmountable racial limits. Although he recognised that freedom and external circumstances significantly influenced mental development, he contended that these effects were ultimately constrained by the inherent intellectual capacities he attributed to each race.

## The quest for recognition: Carus and international scientific racism

Carus was keen to popularise his theory of race among scholars of anatomy and physiology in Europe and North America. Contemporaries took notice of Carus’s works on race, yet the reception remained somewhat muted. Towards the late 1850s, European anatomists increasingly rejected Carus’s theories. Carus, for his part, turned towards the ‘American School’ of Ethnology, which was better aligned with his polygenist ideas, but their reception of his ideas, too, was limited.

Following early accolades for his zoological works, Carus’s works on anatomy were translated into English, French, and Italian in the 1830s.^87^ Subsequently, Carus targeted an international audience in his works on race. He actively promulgated the English translation of his 1844 lecture on the present state of cranioscopy, which was completed by his friend Johann Christian Heinrich Freund in London in the same year.^88^ Two bilingual atlases of cranioscopy were launched in the early 1840s. His Goethe festschrift on racial inequality was republished in the 1860s in the Philadelphia-based magazine *Der Pionier.* ^89^ He kept a meticulous record of reviews of these works in German, English, and French journals.

Like other mid-nineteenth-century craniologists, Carus did not travel extensively to obtain specimens for his collection. His limited international journeys never extended beyond the European heartlands; Austria, England, France, Italy, Scotland, and Switzerland were the sole destinations.^90^ Like his contemporaries Anders Retzius and Samuel George Morton, Carus relied on an extensive network of international contacts that acquired specimens. While Morton relied on diplomats, military personnel, naturalists, and physicians to establish his collection, both Carus and Retzius leveraged their deep connections to the ‘academic anatomical elite of Europe.’^91^ Along with his translations, Carus used these contacts to promote his distinctly *naturphilosophisch* approach to race beyond the German-speaking world.

As his correspondence with fellow anatomists Anders Retzius in Stockholm and Rudolph Wagner in Göttingen shows, the early 1840s provided a window of opportunity for the popularisation of Carus’s racial theories, which ultimately failed to materialise. In August 1841, Carus sent Wagner, custodian of the famous Blumenbach collection in Göttingen, a copy of his treatise on the *Fundamentals of a New and Scientific Cranioscopy* ^92^ (in the following: *Grundzüge*) ‘for your kind perusal.’^93^ Subsequently, although Wagner criticised Carus for attributing mental capacities to the three cerebral masses,^94^ he invited him to Göttingen to revisit Blumenbach’s craniological collection and discuss matters of cerebral physiology in depth – an invitation Carus declined.^95^ Retzius, who received a copy of Carus’s *Atlas of Cranioscopy*, initially attempted to integrate Carus’s association of cranial vertebrae and mental capacities into his work on cerebral anatomy, despite conflicts with his theory that key faculties had to be located in the hemispheres.^96^

As Carus’s work increasingly delved into physiognomy and symbolism in the 1850s, Wagner’s willingness to engage with his craniology seems to have dissipated. Planning a convention of European anthropologists in Göttingen in the late 1850s, Wagner not only described Carus as a ‘fantasist’ who could not satisfy his wish for an empirically founded craniology but insisted on excluding him from the meeting.^97^ Similarly, Retzius lamented to Karl Ernst von Baer that despite his respect for Carus, he could ‘not follow him in many ways.’^98^ In the 1860s, the German anthropologist Carl Vogt, himself an ardent racist, concluded that ‘no one has yet followed’ Carus’s ‘*naturphilosophisch* view to relate three cranial vertebrae to the various mental faculties,’ since ‘the skull bones are so extraordinarily irregular.’^99^

Other naturalists were more welcoming. The Dutch anthropologist and naturalist Jan van der Hoeven devoted a small chapter in his book on the natural history of Black people to the *Grundzüge.* ^100^ He found Carus’s developmental theory regarding the three cranial vertebrae and their correspondence to intellect, feeling, and will plausible. He particularly admired the objectivity in Carus’s skull measurement methodology.^101^ However, for van der Hoewen’s comparative study of Black and white skulls, the *Grundzüge* did not provide enough data since the work only included measurements of two Black skulls.^102^ Lorenz Oken, who had provided the conceptual backdrop for Carus’s vertebrae theory, published favourable reviews of the *Grundzüge* and the *Atlas der Cranioscopie* in his journal *Isis.* While expressing uncertainty about Carus’s localisation of intellect, feeling, and will in specific skull cavities, Oken applauded him for superimposing skull contours of such diverse individuals as Napoleon, Talleyrand, a ‘Greenlander’, and a ‘Cretin’, enabling ‘satisfying comparisons’^103^. Similarly, the British surgeon James Paget published an extensive review of Carus’s physiognomy treatises, praising the proposed correspondences between mind and form. Although race played a minor role in this review, Paget concurred with Carus’s stereotypical observations such as the supposed similarity between ‘the little noses of children or the flat, broad noses of negroes’, agreeing that, when occurring ‘in men of civilised races all such noses indicate defective intellectual power.’^104^

The strongest opposition to Carus’s craniological works came from phrenological circles. In 1842, the *Phrenological Journal* published an extensive review of Carus’s *Grundzüge*, which could not discover a single positive aspect in it. Quite to the contrary, the reviewer dismissed the correspondence between the three cranial vertebrae and the faculties of intellect, feeling, and will as utter nonsense. Without explicitly mentioning Carus’s racial propositions, the critic strongly rebuked the linkage between cranial vertebrae and psychological characteristics, deeming such connections only possible ‘by the most flimsy analogies, the boldest assumptions, and the most confused and indiscriminate use of psychological terms’.^105^ These disparagements resurfaced two years later when the journal reviewed the English translation of Carus’s 1844 lecture on the ‘Present State of Cranioscopy.’ While expressing gratitude to Johann Freund for undertaking the ‘extremely difficult’ task of translating ‘the transcendental quality of both thoughts and style in the original’, the reviewer subjected Carus to a scathing critique. Maintaining that Carus’s ‘opinions’ were ‘based on assumptions, which are contradicted by demonstrable facts’, the reviewer concluded that ‘[a] more thoroughly unscientific production we have rarely perused.’^106^

Carus was aware that his works on craniology had not fared well in the scholarly community, but he attributed this to such readers’ tendency to see either too much or too little of Gall’s phrenology in it.^107^ Perhaps in response to the indifference of European anatomists, Carus increasingly engaged with racial theories from across the Atlantic. The *Atlas of Cranioscopy* not only sought to disseminate Carus’s physiognomics of race beyond borders but was based on the works of one of the founding fathers of scientific racism in America. Samuel George Morton consistently served as a wellspring of inspiration for Carus, who in turn endeavoured to popularise the views of the American naturalist and skull collector within Germany. As Robert J. Richards has shown, Carus’s turn towards cranial physiognomy was triggered by the acquisition of four illustrations from Morton’s seminal racial taxonomy, *Crania Americana*, and his subsequent reading of a German translation of Morton’s writings in 1840.^108^ The woodcuts from *Crania Americana*, drawn by Morton and printed by Philadelphian artist John Collins, provided a blueprint for the visual representations in Carus’s *Atlases.* Unlike Morton, who used a variety of angles, Carus insisted on drawing each skull precisely in profile, rendering the illustrations more objective ‘so that many forms can be compared with each other.’^109^ Notably, Carus’s 1864 *Neuer Atlas der Cranioscopie*, a partial re-edition of his earlier works, also adopted Morton’s style, providing each skull with a title in outline font. Through these choices, Carus’s *Atlases* present themselves as a kind of Saxonian rendition of the *Crania Americana*, strategically addressing some of the latter’s limitations.

In 1842, he had scrutinised these limitations in a comprehensive review of the 1839 English edition of *Crania Americana* authored by Carus for the *Neue Jenaische Allgemeine Literatur-Zeitung.* While he praised Morton’s impressive collection and the book’s rich illustrations, tables, and descriptions, he expressed scepticism regarding Morton’s method of cranial measurement, not least because it diverged from his own. Although Carus commended Morton’s ‘imaginative’^110^ method of filling the skulls with pepper corns to measure volume, he contended that it did not adhere to physiological principles, insisting that only the measurement of the three cranial vertebrae fulfilled such criteria.^111^

Carus’s criticism extended to Morton’s depiction of skulls. While the Philadelphian had devised an apparatus to draw the cranial contours on a glass plate, Carus argued that inherent perspectival distortions misrepresented the dimensions of the skulls. Here, too, Carus advocated for his technique, involving the creation of a clay impression of the skull’s profile, which was then directly transferred onto paper.^112^ Additionally, Carus asserted that the omission of cranial vertebrae had led Morton to ignore the implications of his anatomical data for mental capacity. Aligning himself with Georges Combes, who had authored the afterword of *Crania Americana*, Carus forged Morton’s at times contradictory findings into a coherent white supremacist position.^113^ According to Carus, the small overall size of the skulls of indigenous Americans testified to their ‘lack of natural intelligence’, and their ‘distant relationship to the predator that, without any higher psychic capacities, uses the strength of its senses and muscles to catch and destroy prey.’^114^

Despite these criticisms, Carus’s judgement of Morton’s work was very positive on the whole and he recommended *Crania Americana’s* acquisition to all larger libraries.^115^ In several letters to his friend Regis, Carus reported on the progress he was making with the review, calling *Crania Americana* ‘a great work’.^116^ He hoped that Morton would one day acquire a copy of his handbook on cranioscopy and correct his method of cranial measurement.^117^ There is no surviving correspondence between Carus and Morton, but given the fact that Carus sent copies of his racial treatises to many friends and fellow naturalists, it is conceivable that he actively contributed to the realisation of this dream.^118^ In 1846, Morton donated a copy of the *Atlas der Cranioscopie* to the library of the Academy of Natural Sciences of Philadelphia, which he might have received from Carus.^119^ A decade later, Carus still considered Morton the authority on the racial features of indigenous Americans.^120^

Over the years, Carus’s approval for what would become the ‘American School’ of ethnology only deepened. Carus delved into the works of Morton’s students, George R. Gliddon and Josiah C. Nott, editors of another founding document of scientific racism, the 1854 compilation *Types of Mankind.* ^121^ Carus read this work with great interest and discussed it extensively in a special supplement to *Meyers Konversationslexikon* in 1858.^122^ Here, he first recapitulated the controversy between polygenism and monogenism before endorsing Morton’s polygenist position based on craniological studies.^123^ Even more interesting is Carus’s take on the second ethnological volume edited by Nott and Gliddon, *Indigenous Races of the Earth* (1857).^124^ Carus was particularly interested in one treatise, which he recognised as the most important: ‘The cranial characteristics of the races of men’ by the American physician James Aitken Meigs.^125^ Carus concurred with Meigs that ‘skull formation does not provide evidence of any single sign that distinguishes the main human races, but only the overall habitus.’^126^ While this sounded like a revision of his previous positions, Carus was quick to add that, in his view, cranial dimensions remained ‘the most important indicator for any higher or lower predisposition to spiritual development.’^127^

As in Morton’s case, Carus expressed disappointment that Meigs had not recognised the relevance of the three cranial vertebrae, ‘even though the literature on this subject (as at least his citations show) was by no means foreign to him.’^128^ Intriguingly, this literature turned out to be none other than Carus’s own craniological writings. Despite his admiration for American ethnology, Carus’s exploration of Anglophone racial theories was highly selective, and focused on passages aligning with his ideas. The stance from his 1838 *System of Physiology* remained essentially unaltered over several decades. Rather than using transatlantic racial theories to refine or modify his arguments, Carus employed them to lend further legitimacy to the foundations he had lain. This, in turn, is one possible explanation of why the reception of Carus’s racial theories by Morton and his followers remained limited.

Indeed, among the Anglophone racial theorists featured in Gliddon’s and Nott’s books, Meigs is the only one who referenced Carus’s works. Carus plays a prominent role in this essay alongside the founding fathers of craniology, Blumenbach and Morton; Carus’s manual on cranioscopy supplied ‘to some extent the principles which should guide us in our examination of the different cranial forms, in their relation to psychical conditions.’^129^ In his re-assessment of Morton’s skull collection, Meigs opted to utilise Carus’s depiction of Schiller’s skull, ‘in preference to any of the heads contained in Morton’s Collection, as the standard or typical ideal representative … of Central and Eastern Europe.’^130^ Despite his clear admiration for the German, Meigs stopped short of adopting Carus’s romantic framework of race. Neither the developmental theory of cranial vertebrae nor the diurnal cycle with its corresponding racial characteristics found a place in Meigs’s work. Anglophone polygenists maintained some interest in Carus, and in 1863, he was admitted as a Corresponding Member to the London Anthropological Society, a short-lived polygenist and white supremacist organisation led by James Hunt.^131^ However, a paper he presented to the society on the supposedly animalistic character of the jaw of the Greenlander failed to impress its members as it contained obvious anatomical errors.^132^

In examinations of another pivotal figure in scientific racism, Arthur de Gobineau, historians have noted his references to Carus but arrived at divergent conclusions. Gérard Imhoff contends that Gobineau sought to distance himself from Carus, while Hannes Stubbe positions the Dresden physician as Gobineau’s ideological precursor.^133^ Gobineau did indeed cite Carus’s *Über ungleiche Befähigung* in his *Essai sur l’inegalité des races humaines*, yet he maintained a highly sceptical stance concerning its scientific merit. Above all, Gobineau expressed major doubts about the arbitrary correlation between the diurnal cycle and the four race types, stating that ‘comparison does not amount to reason, and Mr. Carus has somewhat spoiled his beautiful theory by indulging unduly in this poetic approach.’^134^ Evidently, Carus’s romantic framework could not convince even a thinker like Gobineau, who prided himself on constructing a mythological basis for racism.

While Carus engaged extensively with the works of nearly all key figures in the emerging discourses of scientific racism, the same cannot be said in reverse. We posit that the primary factor limiting the reception of Carus’s ideas, even among like-minded racial scientists, was the waning appetite for *Naturphilosophie* by the 1830s to 1850s, as can be seen in Carus’s increasing alienation from anatomists like Retzius and Wagner. Although he shared the inherent racist convictions of mid-nineteenth-century racial science, especially across the Atlantic, promulgating the superiority of white bodies and minds over those of non-whites, the metaphysical foundation of this superiority became increasingly perplexing to anthropologists and craniologists in the 1840s and 1850s. Carus’s reliance on the diurnal cycle as the guiding principle of racial hierarchy, coupled with the association of race with the vertebrate theory of the skull rested on a *naturphilosophisch* style of reasoning that no longer convinced. Even scientists sympathetic towards Carus’s physiognomy of race acknowledged that, regarding the ‘correspondence between mind and form … much more remains to be accomplished by minds of a less imaginative tendency than that of Carus.’^135^

## Conclusion

This analysis of Carus’s anatomical, anthropological, cranioscopical, and other writings, together with unpublished material, has demonstrated that race and human inequality held a central place in his oeuvre. Although Carus’s *naturphilosophisch* framework presented obstacles for the adoption of his ideas by fellow racial theorists, some selectively adapted aspects of his work, particularly regarding the physiognomy of race. Carus, in turn, exhibited a keen awareness of the evolving discourses of scientific racism in the 1840s and 1850s. Notably, his interest was piqued by racial theories emerging from North America. He held the works of Samuel Morton, George Gliddon, and Josiah Nott in high esteem. Morton’s *Crania Americana* provided the blueprint for Carus’s cranioscopical works; both sought to establish an anatomical basis for racial inequality, and both harboured an implicit methodological bias in their selection of sample skulls. Arthur de Gobineau, by contrast, played a comparatively minor role in shaping and sustaining Carus’s racist agenda. This might be attributed to Carus’s theory of race, rooted in anti-Blackness and dehumanising sentiments towards the indigenous people of the Americas. Throughout his writings, Carus frequently deployed notions of racial inferiority as an apology for both the Atlantic slave trade and the colonisation of the Americas. In this way, Carus’s theory of race seems more closely aligned with contemporary discourses of settler colonialism than with the theories that laid the ideological foundation for racism in Nazi Germany – an intellectual genealogy previously suggested by some historians.

Nevertheless, it is worth noting that several of Carus’s works, including his major treatise on race *On the Unequal Capacity of Different Races* were republished during the Nazi era. The demand for pseudo-scientific justifications of racism during that time may have facilitated the resurgence of his writings. However, more biologically oriented racial theorists had little use for Carus’s theories, which they deemed ‘too far removed from the insights that are essential to us today.’^136^ Moreover, Carus’s categorisation of Jews as people of the day was also incompatible with the profound anti-Semitism of Nazi ideology. However, it remains the case that Carus, often praised as a universal genius with a humanist stance, besides his achievements, helped to create the theoretical justification for the dehumanisation of large parts of the human population.

## Footnotes

1

Berndt Lindfors , Ira Aldridge: Performing Shakespeare in Europe, 1852–1855 (Rochester: University of Rochester Press, 2013), 148 .

2

Carl Gustav Carus , Lebenserinnerungen und Denkwürdigkeiten, 5 vols (Leipzig: Brockhaus, Vol. 1–4; Dresden: Jeß, Vol. 5, 1865–1931), Vol. 2, 268–9.

3
*Ibid.*, Vol. 2, 269.

4

Michael Hagner , ‘The Soul and the Brain between Anatomy and Naturphilosophie in the Early Nineteenth Century’, Medical History, 36, 1 (1992), 1–33, 51542232 10.1017/s0025727300054600PMC1036519.

5

Heinz-Egon Kleine-Natrop , Das heilkundige Dresden: Dresdner Chirurgenschulen und medizinische Lehrstätten in drei Jahrhunderten (Leipzig, Dresden: Th. Steinkopff, 1964), 127–53. Wolfgang Genschorek , Carl Gustav Carus: Arzt, Künstler, Naturforscher, 4th edn (Leipzig: S. Hirzel, 1986); Dietrich von Engelhardt , Medizin in Romantik und Idealismus, 4 vols (Stuttgart, Bad Cannstatt: frommann-holzboog Verlag, 2023), Vol. 3, 52–97 .

6 Kleine-Natrop, *op. cit.* (note 5), 133; Caris-Petra Heidel, ‘“…von Wissenschaft keine Spur(?): Rezeption und zeitgenössische Kritik des medizinischen und naturkundlichen Werkes von Carl Gustav Carus”’, in Petra Kuhlmann-Hodick, Spitzer-Gerd and Bernhard Maaz (eds), *Carl Gustav Carus: Wahrnehmung und Konstruktion* (Berlin, München: Deutscher Kunstverlag, 2009), 73–81, 75.

7

Richard T. Gray , About Face: German Physiognomic Thought from Lavater to Auschwitz (Detroit: Wayne State University Press, 2004), 133–6.

8 Heidel, *op. cit.* (note 6), 73–81, 78–9.

9

Carl Gustav Carus , Ueber ungleiche Befähigung der verschiedenen Menschheitstämme für höhere geistige Entwicklung (Leipzig: F. A. Brockhhaus, 1849).

10 Carl Gustav Carus, Symbolik der menschlichen Gestalt (Leipzig: F. A. Brockhaus, 1853). For a selection of scholars focusing on these two works in their analyses of Carus’s racism, see Hannes Stubbe , ‘Hatten die Germanen graue Augen? Rassenpsychologisches bei C.G. Carus’, Psychologie und Geschichte, 1, 3 (1989), 44–53 ; Jutta Müller-Tamm , Kunst als Gipfel der Wissenschaft: Ästhetische und wissenschaftliche Weltaneignung bei Carl Gustav Carus (Berlin, New York: De Gruyter, 1995), 113–137 ; Gray, About Face, *op. cit.* (note 7), 99–136; Jana Husmann , Schwarz-Weiss-Symbolik: Dualistische Denktraditionen und die Imagination von ’Rasse’ (Bielefeld: Transcript-Verlag, 2010), 183–228 .

11 Stubbe, *op. cit.* (note 10), 45–6; George L. Mosse, *Toward the Final Solution: A History of European Racism*, with a critical introduction by Christopher R. Browning (Madison, Wisconsin: The University of Wisconsin Press, 2020 [1978]), 28–9.

12

Michael Hagner , ‘Zur Physiognomik des Geistes bei Carl Gustav Carus’, in Petra Kuhlmann-Hodick , Spitzer-Gerd and Bernhard Maaz (eds), Carl Gustav Carus: Wahrnehmung und Konstruktion (Berlin, München: Deutscher Kunstverlag, 2009), 261–9; Kathleen Melzer, ‘Die Cranioskopische und Chirognomische Sammlung des Geheimen Rath Dr. C. G. Carus’, in *ibid.*, 253–60.

13 Müller-Tamm, *op. cit.* (note 10), 121.

14
*Ibid.*, 268.

15

Stephen J. Gould , The Mismeasure of Man, 2nd rev (New York: Norton, 1996), 86 .

16 Müller-Tamm, *op. cit.* (note 10), 130.

17 Two scholars claim that Carus’s engagement with racial classification began in 1835; the first published work, however, is from 1838. Michael Banton , Racial Theories, 2nd edn (Cambridge: Cambridge University Press, 2002), 35 ; Herbert Bräutigam , ‘Zu kulturphilosophischen Positionen von Carl Gustav Carus’, Abhandlungen und Berichte des Staatlichen Museums für Völkerkunde Dresden, 44 (1990), 133–40, 133.

18 Müller-Tamm, *op. cit.* (note 10), 135; Richard T. Gray , ‘Physiognomik im Spannungsfeld zwischen Humanismus und Rassismus: Johann Caspar Lavater und Carl Gustav Carus’, Archiv für Kulturgeschichte, 81, 2 (1999), 313–38; Hagner, ‘Zur Physiognomik des’, *op. cit.* (note 12), 262–3.

19

Carl Gustav Carus , System der Physiologie, 3 vols (Dresden, Leipzig: Gerhard Fleischer, 1838-1840).

20
*Ibid.*, I, 122.

21
*Ibid.*, Vol. 1, 126–7.

22

Carl Gustav Carus , Atlas der Cranioscopie oder Abbildungen der Schädel- und Antlitzformen berühmter oder sonst merkwürdiger Personen, 2 vols (Leipzig: August Weichardt, 1843-1845); Carl Gustav Carus , Neuer Atlas der Cranioskopie enthaltend dreissig Tafeln Abbildungen merkwürdiger Todtenmasken und Schädel (Leipzig: F. A. Brockhaus, 1864).

23

Carl Gustav Carus , Grundzüge einer neuen und wissenschaftlich begründeten Cranioscopie (Schädellehre) (Stuttgart: Balz’sche Buchhandlung, 1841).

24

Carl Gustav Carus , Über Grund und Bedeutung der verschiedenen Formen der Hand in verschiedenen Personen (Stuttgart: Ad. Becher’s Verlag, 1846), 5–6 .

25

Carl Gustav Carus , ‘Die Frage nach Entstehung und Gliederung der Menschheit vom Standpunkte gegenwärtiger Forschung’, Unsere Zeit, 2 (1858), 65–97 .

26

Carl Gustav Carus , Natur und Idee oder das Werdende und sein Gesetz (Wien: Wilhelm Braumüller, 1861).

27 Carus, *Ueber ungleiche Befähigung, op. cit.* (note 9), 27.

28
*Ibid.*, 29.

29
*Ibid.*, 83.

30 Carus, *Lebenserinnerungen und Denkwürdigkeiten*, *op. cit.* (note 2); Carl Gustav Carus , England und Schottland im Jahre 1844, 2 vols (Berlin: Duncker, 1845); Carl Gustav Carus , C. G. Carus an Regis: eine Brieffolge von 1814 – 1853, 3 vols (Leipzig: 1956).

31 Carus, *England und Schottland, op. cit.* (note 30), Vol. 1, 290–1.

32 Dietrich von Engelhardt, ‘Morphologie in der metaphysischen Naturphilosophie und romantischen Naturforschung und Medizin um 1800’, in Laura Follesa and Federico Vercellone (eds), *Bilddenken und Morphologie* (De Gruyter, 2021), 99–114, 108–9.

33

Carl Gustav Carus , Versuch einer Darstellung des Nervensystems und insbesondre des Gehirns nach ihrer Bedeutung, Entwickelung und Vollendung im thierischen Organismus (Leipzig: Breitkopf und Härtel, 1814); Carl Gustav Carus , Von den Ur-Theilen des Knochen- und Schalengerüstes (Leipzig: Gerhard Fleischer, 1828).

34 Carus, *Versuche einer Darstellung*, *op. cit.* (note 33), 310–311.

35
*Ibid.*, 272.

36
*Ibid.*, 274–5.

37
*Ibid., 312;* See also Hagner, ‘The Soul and the Brain’, *op. cit.* (note 4), 30.

38 Carus, *Versuche einer Darstellung*, op. cit. (note 33), 311.

39 Susanne Lettow, ‘Modes of naturalization: Race, sex, and biology in Kant, Schelling and Hegel’, Philosophy & Social Criticism, 39, 2 (2013), 117–31, 124; Owsei Temkin , ‘German concepts of ontogeny and history around 1800’, Bulletin of the History of Medicine, 24, 3 (1950), 227–46, 24414792164 ; Hans Vermeulen , ‘The German Invention of *Völkerkunde*: Ethnological Discourse in Europe and Asia, 1740-1798,’ in Sara Eigen and Mark Larrimore , eds., The German Invention of Race (Albany: State University of New York Press, 2006), 123–45, 133–5.

40
*Ibid.*

41
*Ibid.*, 312.

42 Carus, *Von den Ur-Theilen, op. cit.* (note 33).

43 Nicolaas A. Rupke, *Richard Owen: Biology without Darwin*, 2nd edn, rev (Chicago: The University of Chicago Press, 2009 [1994]), 124.

44 Philip F. Rehbock, ‘Transcendental Anatomy’, in Andrew Cunningham and Nicholas Jardine (eds), Romanticism and the Sciences (Cambridge: Cambridge University Press, 1990), 144–60, 146.

45

Robert J. Richards , The Romantic Conception of Life: Science and Philosophy in the Age of Goethe (Chicago, London: Chicago University Press, 2002), 443 .

46
*Ibid.*, 493.

47 Robert J. Richards, ‘Romantic Biology: Carl Gustav Carus at the Edge of the Modern’, in Elizabeth Millán Brusslan (ed.), The Palgrave Handbook of German Romantic Philosophy (London: Palgrave Macmillan, 2020), 347–74, 363.

48 Carus, *Von den Ur-Theilen*, *op. cit.* (note 33), 176.

49

Carl Gustav Carus , Paris und die Rheingegenden, Vol. 1 (Leipzig: Gerhard Fleischer, 1836), 180 . See also Sadiah Qureshi , ‘Displaying Sara Baartman, the “Hottentot Venus,”’ History of Science 42, 2 (2004), 233–57.

50

Carl Gustav Carus , Paris und die Rheingegenden, Vol. 2, (Leipzig: Gerhard Fleischer, 1836), 275 .

51

Kathleen Melzer , ‘Die Schädel- und Abgusssammlung von Carl Gustav Carus’, in Petra Kuhlmann-Hodick , Spitzer-Gerd and Bernhard Maaz (eds), Carl Gustav Carus: Wahrnehmung und Konstruktion (Berlin, München: Deutscher Kunstverlag, 2009), 317–23, 317 .

52 Carl Gustav Carus, ‘Vom Leben der Menschheit: Bruchstück aus einer künftig erscheinenden Physiologie’, *Zeitschrift für Philosophie und speculative Theologie*, 2, 1 (1838), 1–20. Carus, *Versuch einer Darstellung des Nervensystems*, *op. cit.* (note 33), 301.

53

Johann Gottfried Herder , Ideen zur Philosophie der Geschichte der Menschheit, Vol. 2 (Riga, Leipzig: Johann Friedrich Hartknoch, 1785), 77 .

54 Carus, *System der Physiologie, op. cit.* (note 19), Vol. 1, 118–9.

55 Herder, *Ideen zur Philosophie*, Vol. 2, *op. cit.* (note 53), 81.

56

Nicolaas A. Rupke , ‘The Origins of Scientific Racism and Huxley’s rule’, in Nicolaas A. Rupke and Gerhard Lauer (eds), Johann Friedrich Blumenbach: Race and Natural History, 1750–1850 (Abingdon, Oxon, New York, NY: Routledge, 2018), 223–47, 239.

57 Carus, *System der Physiologie*, *op. cit.* (note 19), Vol. 1, 118–9. This concept of human development, from primordial non-Europeans into more perfected Europeans, echoed a common racist bias of late eighteenth-century anthropology, such as Immanuel Kant’s. Gayatri C. Spivak, A Critique of Postcolonial Reason: Toward a History of the Vanishing Past (Cambridge, Mass., London: Harvard University Press, 1999), 27.

58 Carus, *System der Physiologie*, Vol. 1, *op. cit.* (note 19), 109–113.

59 Carus, ‘Die Frage nach Entstehung’, *op. cit.* (note 25), 95–8.

60 Carus, *System der Physiologie*, *op. cit.* (note 19), Vol. 1, 125.

61

Joris van Gorkom , ‘Skin Color and Phlogiston: Immanuel Kant’s Racism in Context’, History of Philosophy of the Life Sciences, 42, 2 (2020)10.1007/s40656-020-00311-432347394.

62 Carus, *System der Physiologie*, *op. cit.* (note 19), Vol. 1, 125.

63
*Ibid.*

64
*Ibid.*

65
*Ibid.*, 127.

66 W. E.B. Du Bois, ‘The Souls of White Folk’, in *Darkwater* (London: Verso, 2016 [1920]), 17–29, at p. 24.

67 Anonymous, *Vorbereitung zu des Herrn Dr. Gall Vorlesungen über die Cranioscopie: Von einem seiner Schüler* (Leipzig: Schäferische Buchhandlung, 1805).

68 Carus, *Grundzüge einer neuen und wissenschaftlich begründeten Cranioscopie*, *op. cit.* (note 23), 9.

69 Carus, *System der Physiologie, op. cit.* (note 19), Vol. 3, 115–16.

70 Carus, *Grundzüge einer neuen und wissenschaftlich begründeten Cranioscopie*, *op. cit.* (note 23), 11.

71 In this vein, Carus refuted Blumenbach’s examples of prominent Black men who had achieved intellectual merits. Carus, *Ueber ungleiche Befähigung der verschiedenen Menschheitstämme*, *op. cit.* (note 9), 24.

72 Mario Marino, ‘Naturgeschichte und Rassenklassifikation: Zu Blumenbachs Anthropologie und ihrer Rezeption’, in Laura Follesa and Federico Vercellone (eds), *Bilddenken und Morphologie* (De Gruyter, 2021), 73–98, 89.

73 Carus, *System der Physiologie*, *op. cit.* (note 19), Vol. 1, 126. For Blumenbach’s attribution of Caucasian skulls as ‚beautiful‘, see Robert J. Richards , ‘The Beautiful Skulls of Schiller and the Georgian Girl: Quantitative and Aesthetic Scaling of Races, 1770–1850’, in Nicolaas A. Rupke and Gerhard Lauer (eds), Johann Friedrich Blumenbach: Race and Natural History, 1750–1850 (Abingdon, Oxon, New York, NY: Routledge, 2018), 142–75.

74 Carus, *System der Physiologie, op. cit.* (note 19), Vol. 1, 126.

75 Carus, *Atlas der Cranioscopie*, *op. cit.* (note 22), Vol. 1, unpaginated.

76 The skull was taken from a mass grave, where not only Schiller, but 20 other bodies were buried. Wulf D. Hund, ‘Die Körper der Bilder der Rassen: Wissenschaftliche Leichenschändung und rassistische Entfremdung’, in *idem* (ed.), *Entfremdete Körper: Rassismus als Leichenschändung* (Bielefeld: Transcript-Verlag, 2009), 13–80, 13–4.

77

Hermann Welcker , Schillers Schädel und Totenmaske (Braunschweig: Vieweg, 1883), 40 .

78
*Ibid.*

79 Carus, *System der Physiologie*, *op. cit.* (note 19), Vol. 1, 149.

80
*Ibid.*, 149.

81
*Ibid*, 130.

82
*Ibid*, 149.

83 Carus, *Ueber ungleiche Befähigung der verschiedenen Menschheitstämme, op. cit.* (note 9), 83.

84 Sächsische Landesbibliothek – Staats- und Universitätsbibliothek Dresden, Mscr e 86m: Carl Gustav Carus, *Vom Urbilde des Staates* (1850), 23v r.

85
*Ibid.*, 5v.

86 Johann M. Leupoldt, ‘Carl Gustav Carus: System der Physiologie’*, Jahrbücher für wissenschaftliche Kritik*, 2 (1840), Sp. 61-75, Sp. 69.

87 Carl Gustav Carus, An Introduction to the Comparative Anatomy of Animals with Compiled Reference to Physiology, and Elucidated by Twenty Copperplates, R. T. Gore (trans.), 3 vols (London: Longman, 1827); Carl Gustav Carus , Traité élémentaire d’anatomie comparée suivi de recherches d’anatomie philosophique ou transcendante sur les parties primaires du système nerveux et du squelette intérieur et extérieur, A.-J.-L. Jourdan (trans.) (London, Paris: J.-P. Baillière, 1835); Carl Gustav Carus , Atlas d’anatomie comparée, A.-J.-L. Jourdan (trans.) (Brussels: H. Dumont, 1836).

88 Carl Gustav Carus, ‘On the Present State of Cranioscopy: A Lecture Delivered at Leipzig, on the 3rd of February 1844’, Translated from the German by J. C. H. Freund, *London Medical Gazette*, 2, August 8 (1844).

89 Carus, *Lebenserinnerungen und Denkwürdigkeiten*, *op. cit.* (note 2), Vol. 3, 293.

90 Genschorek, *op. cit.* (note 5), 86, 138, 160, 183.

91 Olof Ljungström, ‘Swedish Golgotha: Anders Retzius’ collection of human crania at the Karolinska Institutet according to an 1862 manuscript’, Lychnos (2014), 159–72, 171. For Morton’s donors, see: Ann Fabian, The skull collectors: Race, science, and America’s unburied dead (Chicago: The University of Chicago Press, 2010). The composition of Carus’s craniological network has been reconstructed from his correspondence, which is held at TU Dresden’s University Library. It included many prominent figures of early anthropology, such as Karl von Baer, Joseph Barnard Davis, Daniel Frederik Eschricht, Johann Christian Gustav Lucae, Anders Retizus, John Thurnam, and Rudolph Wagner. Sächsische Landesbibliothek – Staats- und Universitätsbibliothek Dresden, Mscr.Dresd.h.45, Bd.1–6. Since the details of this network exceed the limits of this article, the authors intend to publish a more detailed account of Carus’s craniological network in a separate paper.

92 Carus, *Grundzüge einer neuen und wissenschaftlich begründeten Cranioscopie, op. cit.* (note 23).

93 Universitäts- und Landesbibliothek Göttingen, Cod. Ms. R. Wagner II: Carl Gustav Carus to Rudolf Wagner, 27 August, 1841.

94 Rudolph Wagner, *Handwörterbuch der Physiologie: mit Rücksicht auf physiologische Pathologie* (Braunschweig: Friedrich Vieweg und Sohn, 1842), 586.

95 Niedersächsische Staats- und Universitätsbibliothek Göttingen, Cod. Ms. R. Wagner II: Carl Gustav Carus to Rudoloph Wagner, June 13th, 1843.

96 Sächsische Landesbibliothek – Staats- und Universitätsbibliothek Dresden, Mscr.Dresd.h.45.Bd.5, Nr. 138: Anders Retzius to Carl Gustav Carus, July 3rd, 1845.

97 Universitätsbibliothek Gießen, Nachl. Baer, Briefe 20, fol. 285–286: Rudolf Wagner to Karl Ernst von Baer, March 3rd, 1861, fol. 285r.

98 Universitätbibliothek Gießen, Nachl. Baer, Briefe 17: Anders Retzius to Karl Ernst v. Baer, March 7th, 1859, fol. 672r.

99 Carl Vogt, *Vorlesungen über den Menschen*, Vol. 1 (Gießen: J. Ricker’sche Buchhandlung, 1863), 52.

100

Jan van der Hoeven , Bijdragen tot de natuurlijke geschiedenis van den negerstam (Leiden: S. en J. Luchtmans, 1842), 37–41 .

101
*Ibid.*, 37–8.

102
*Ibid.*, 40–1.

103 Lorenz Oken, ‘Review: Atlas der Cranioscopie’, *Isis: encyclopädische Zeitschrift, vorzügl. für Naturgeschichte, vergleichende Anatomie u. Physiologie* (1844), Col. 461–Col. 462, Col. 462.

104 James Paget, ‘Physiognomy of the Human Form’, *The Quarterly Review*, 99, September (1856), 452–91, 477.

105

Anonymous, ‘Review: Dr Carus’s Principles of a New and Scientific Cranioscopy’, Phrenological Journal and Magazine of Moral Science, 15, 71 (1842), 154–60, 159.

106

Anonymous, ‘Review: On the Present State of Cranioscopy’, Phrenological Journal and Magazine of Moral Science, 17 (1844), 408–9, 408.

107 Carus, *Symbolik der menschlichen Gestalt*, *op. cit.* (note 10), XIII–XX.

108 Richards, ‘The Beautiful Skulls’, *op. cit.* (note 73), 164.

109 Carus, *Atlas der Cranioscopie, op. cit.* (note 22), Vol. 1, unpaginated.

110 Carl Gustav Carus, ‘Samuel George Morton: Crania americana’, *Neue Jenaische Allgemeine Literatur-Zeitung*, 1, 267-269 (1842), 1100–8, 1104.

111
*Ibid.*, 1106.

112
*Ibid.*, 1107.

113

Christopher Heaney , Empires of the Dead: Inca Mummies and the Peruvian Ancestors of American Anthropology (New York, NY: Oxford University Press, 2023), 121–2.

114 Carus, ‘Samuel George Morton’, *op. cit.* (note 110), 1107.

115
*Ibid.*, 1108.

116 Carus, *Carus an Regis*, *op. cit.* (note 30), Vol. 3, fol. 507r, 508r, 519r, 520r, 521r.

117 Carus, *Grundzüge einer neuen und wissenschaftlich begründeten Cranioscopie, op. cit.* (note 23), 15–16, note 1.

118 For example, Carus sent a copy of his Morton review to Gottlob Regis, a copy of his article on the development and organisation of mankind to Karl Ernst van Baer via Karl Julius Fritsche, and copies of craniological works, the symbolism of the human body, and Greek skulls to Alexander von Humboldt. Carus, *C. G. Carus an Regis, op. cit.* (note 30), Vol. 3, fol. 521r; Carl Gustav Carus to Karl Julius Fritsche, 11 December 1858, in: Nachlass Karl Ernst von Baer, Briefe IV, https://digisam.ub.uni-giessen.de/ubg-ihd-nl-baer-k/content/titleinfo/3890516 (accessed 20 February 2024); Ingo Schwarz , ‘Carl Gustav Carus und Alexander von Humboldt: Briefwechsel’, in Ottmar Ette and Eberhard Knobloch (eds), HiN: Alexander von Humboldt im Netz (Potsdam: Universitätsverlag Potsdam, 2009), 5–29, 17, 18, 26.

119 ‘Stated Meeting, August 4, 1846’, *Proceedings of the Academy of Natural Sciences of Philadelphia*, 3, 4 (1846), 89–92, 90.

120 Carl Gustav Carus, ‘Die sogenannten Aztekenkinder [2]’, *Dresdner Journal*, 36 (1856).

121

Josiah C. Nott and George R. Gliddon (eds), Types of Mankind: Ethnological Researchers based upon the Ancient Monuments, Paintings, Sculptures, and Crania of Races and upon their Natural, Geographical, Philological, and Biblical History (London: Trübner, 1854).

122 Carus, ‘Die Frage nach Entstehung’, *op. cit.* (note 25), 66–81.

123
*Ibid.*, 67–8.

124

Josiah C. Nott and George R. Gliddon (eds), Indigenous Races of the Earth: New Chapters of Ethnological Enquiry (London: Trübner, 1857).

125

James A. Meigs , ‘The Cranial Characteristics of the Races of Men’, in Josiah C. Nott and George R. Gliddon (eds), Indigenous Races of the Earth: New Chapters of Ethnological Enquiry (London: Trübner, 1857), 203–352 .

126 Carus, ‘Die Frage nach Entstehung’, *op. cit.* (note 25), 85.

127
*Ibid.*

128
*Ibid.*, 84.

129 Meigs, *op. cit.* (note 125), 217.

130
*Ibid.*, 303.

131 Sächsische Landesbibliothek – Staats- und Universitätsbibliothek Dresden, Mscr.Dresd.h.45, Bd. 3, Nr. 89: Alfred Higgins to Carl Gustav Carus, August 19th, 1863. Ronald Rainger, ‘Race, Politics, and Science: The Anthropological Society of London in the 1860s’, *Victorian Studies*, 22, 1 (1978), 51–70.

132 Charles C. Blake, ‘Report on “Some Remarks on the Construction of the Upper Jaw of the Skull of a Greenlander presented by C. G. Carus”’, *Journal of the Anthropological Society*, 2 (1864), CXIV–CXV.

133 Gérard Imhoff, ‘Charles Gustave Carus, precurseur de Gobineau?’, *Etudes gobiniennes*, 3 (1968-1969), 195–205, 205; Stubbe, *op. cit.* (note 10), 45.

134

Arthur de Gobineau , Essai sur l’inégalité des races humaines (Paris: Firmin-Didot frères, 1853), 189 .

135 Paget, *op. cit.* (note 104), 490–1.

136

Hans Kern , Carl Gustav Carus: Persönlichkeit und Werk (Berlin: Widukind-Verlag, 1942), 170 .

